# Effects of Intrauterine Inflammation on Cortical Gray Matter of Near-Term Lambs

**DOI:** 10.3389/fped.2018.00145

**Published:** 2018-06-15

**Authors:** Vanesa Stojanovska, Anzari Atik, Ilias Nitsos, Béatrice Skiöld, Samantha K. Barton, Valerie A. Zahra, Karyn Rodgers, Stuart B. Hooper, Graeme R. Polglase, Robert Galinsky

**Affiliations:** ^1^The Ritchie Centre, Hudson Institute of Medical Research, Clayton, VIC, Australia; ^2^Department of Women's and Children's Health, Karolinska Institute, Stockholm, Sweden; ^3^Centre of Clinical Brain Sciences, University of Edinburgh, Edinburgh, United Kingdom; ^4^Department of Obstetrics and Gynaecology, Monash University, Clayton, VIC, Australia

**Keywords:** chorioamnionitis, intrauterine inflammation, gray matter, ventilation, neuronal loss

## Abstract

**Introduction:** Ventilation causes cerebral white matter inflammation and injury, which is exacerbated by intrauterine inflammation. However, the effects on cortical gray matter are not well-known. Our aim was to examine the effect of ventilation on the cerebral cortex of near-term lambs exposed to intrauterine inflammation.

**Method:**Pregnant ewes at 119 ± 1 days gestation received an intra-amniotic injection of saline or lipopolysaccharide (LPS; 10 mg). Seven days later, lambs were randomized to either a high tidal volume injurious ventilation strategy (INJ_SAL_ *N* = 6, INJ_LPS_ *N* = 5) or a protective ventilation strategy (PROT_SAL_ *N* = 5, PROT_LPS_ *N* = 6). Respiratory parameters, heart rate and blood gases were monitored during the neonatal period. At post-mortem, the brain was collected and processed for immunohistochemical assessment. Neuronal density (NeuN), apoptotic cell death (caspase 8 and TUNEL), microglial density (Iba-1), astrocytic density (GFAP), and vascular protein extravasation (sheep serum) were assessed within the frontal, parietal, temporal and occipital lobes of the cerebral cortex.

**Results:**A significant reduction in the number of neurons in all cortical layers except 4 was observed in LPS-exposed lambs compared to controls (layer #1: *p* = 0.041; layers #2 + 3: *p* = 0.023; layers #5 + 6: *p* = 0.016). LPS treatment caused a significant increase in gray matter area, indicative of edema. LPS+ventilation did not cause apoptotic cell death in the gray matter. Astrogliosis was not observed following PROT or INJ ventilation, with or without LPS exposure. LPS exposure was associated with vascular protein extravasation.

**Conclusion:**Ventilation had little effect on gray matter inflammation and injury. Intrauterine inflammation reduced neuronal cell density, caused edema of the cortical gray matter, and blood vessel extravasation in the brain of near-term lambs.

## Introduction

Preterm birth (defined as < 37 weeks gestation) affects approximately 15 million births worldwide annually, and is a major cause of neonatal morbidity and mortality (1, 2). Preterm infants have underdeveloped lungs, thus, there is an increased requirement for respiratory support in the delivery room. Whilst respiratory support is a life-saving intervention, manual or automated resuscitation techniques are poorly controlled, and this can lead to injurious effects on the pulmonary, cardiovascular, and cerebral systems (3–6). Recent studies have shown that the initiation of respiratory support causes systemic inflammation and haemodynamic instability resulting in brain inflammation and injury (5, 7). This condition, termed “ventilation-induced brain injury” (VIBI), is associated with diffuse white matter gliosis and compromised blood brain barrier integrity (8). Importantly, improving ventilation in the delivery room can reduce markers of brain inflammation and injury (7, 9). However, these studies have focused extensively on the white matter. The acute effect of ventilation on cortical gray matter in preterm neonates remains unknown. This is a significant gap in research, given the vulnerability of gray matter to injury in the near-term brain (10). Injury to the cortical gray matter can contribute to cognitive and physical disability such as cerebral palsy, a neurodevelopmental disorder commonly associated with preterm birth and intrauterine infection and inflammation (11, 12).

Furthermore, approximately 60% of preterm neonates are exposed to chorioamnionitis and display an increased risk and severity of neurological complications (9, 13–17). There is a clear association between white matter injury following preterm birth and intrauterine infection and inflammation (7, 14, 18–22). We previously showed that improving the ventilation strategy in lambs exposed to intrauterine inflammation, via intra-amniotic LPS, did not reduce VIBI in the white matter (7). However, little is known about the effects on the cortical gray matter.

We aimed to assess the effects of ventilation on the cortical gray matter, and to determine whether intrauterine inflammation exacerbates cortical gray matter inflammation and injury. The second aim was to determine whether a protective ventilation strategy could mitigate markers of inflammation and injury in the cortical gray matter in preterm lambs, in the presence or absence of intrauterine inflammation.

## Materials and methods

### Ethical approval

The experimental protocol was conducted according to the guidelines established by the National Health and Medical Research Council of Australia and was approved by the Monash Medical Centre animal ethics committee (Animal Ethics #MMCA-2015-37).

### Preterm delivery and ventilation

Ultrasound guided intra-amniotic (IA) injection of Lipopolysaccharide (LPS) (10 mg/d; from *Escherichia coli* 055:B5; Sigma-Aldrich, Australia) or saline was administered in Border Leicester ewe's (sourced from Monash University, Churchill) at 119 ± 1 days of gestation (term~148 days; *N* = 5–6/group). Successful IA placement of injection was confirmed with electrolyte analysis of a sample of amniotic fluid (23).

At 126 ± 1 days, pregnant ewes were anesthetized with intravenous (IV) sodium thiopentane, and inhalation of isoflurane (1.5–3.0% in 100% oxygen, Bomac Animal Health, NSW, Australia), and underwent cesarean section. Lambs were exposed, and polyvinyl catheters containing heparinised saline were placed into a jugular vein and carotid artery, for infusion of analgesia and for withdrawing blood for blood-gas analysis. Lambs were intubated (cuffed 4–4.5 mm) then randomly assigned to receive either a “protective ventilation” strategy (PROT_SAL_, *N* = 5 or PROT_LPS_, *N* = 6) or an “injurious ventilation” strategy (INJ_SAL_, *N* = 6 or INJ_LPS_, *N* = 5) as described previously (9).

Briefly, the “protective ventilation' strategy included prophylactic surfactant (100 mg/kg, Curosurf, Chiesi Pharma, Italy), one sustained inflation for 30 s with a peak inflation pressure (PIP) of 35 cmH_2_O (Neopuff; Fisher and Paykel Healthcare, Panmure, Auckland, New Zealand), followed by ventilation (Babylog 8,000+; Dräger, Lübeck, Germany) using volume guarantee mode with a set tidal volume (V_T_) of 7 mL/kg, and a positive end expiratory pressure (PEEP) of 5 cmH_2_O for 90 min. The “injurious ventilation' strategy targeted a V_T_ of 10–12 mL/kg for the first 15 min, with 0 PEEP, with a max PIP set at 50 cmH_2_O to prevent pneumothoraxes. At 15 min lambs in the injury group were placed on volume guarantee mode with a V_T_ of 7 mL/kg, and a PEEP of 5 cmH_2_O for the remainder of the ventilation period. The fraction of inspired oxygen was initially set at 0.4 in both groups and then subsequently adjusted to maintain arterial oxygen saturation (SaO_2_) between 88 and 95%. Respiratory rate was adjusted to maintain partial pressure of carbon dioxide (PaCO_2_) between 45 and 55 mmHg. Lamb well-being was monitored by frequent arterial blood gas measurement (ABL30, Radiometer, Copenhagen, Denmark). Ventilator parameters, including PIP, mean airway pressure (PAW) and V_T_ were recorded in real time (PowerLab; ADInstruments, Castle Hill, NSW, Australia). Physiological parameters including arterial oxygen saturation, heart rate (Massimo, Irvine, CA) and cerebral oxygenation (SctO2, by Near Infrared Spectroscopy: Casmed, USA) were similarly recorded.

### Tissue collection

After 120 min of ventilation, lambs were euthanized with an overdose of sodium pentobarbitone (100 mg/kg IV). Brains from the PROT_SAL_ and INJ_SAL_ groups were transcardially perfused *in situ* with isotonic saline and 4% paraformaldehyde in 0.1 M phosphate buffer (PFA; pH 7.4) and left in fixative overnight. The brain was halved and then cut coronally into 5 mm thick blocks. Blocks of the right cerebral hemisphere were then further fixed in 4% PFA (4 days, 4°C) and embedded in paraffin. Brains from the PROT_LPS_ and INJ_LPS_ groups were excised, halved along the midline and the right cerebral hemisphere was immersion fixed in 4% PFA (overnight, 4°C). The hemisphere was then cut coronally into 5 mm thick blocks and further fixed in 10% PFA (4 days; 4°C).

### Immunohistochemistry

Ten Micrometer sections from equivalent sites from each lobe (frontal, parietal, temporal and occipital) of the right cerebral hemisphere (four sections/animal) were reacted with the following antibodies: mouse anti-NeuN (1:200, MAB377; Millipore) to identify neural nuclei, rabbit anti-caspase 8 (1:100, orb10241; Biorbyt, USA) to label for apoptotic cell death, rabbit anti-Iba-1 (1:1,500, 019-19741; WAKO Pure Chemical Industries, Osaka, Japan) to identify microglia, rabbit anti-GFAP (1:1,000, Zo2334; DAKO, Carpinteria, CA) to identify astrocytes, and rabbit anti-serum albumin (1:1,000, Accurate Chemical and Scientific Corporation, USA) to assess blood brain barrier permeability. All sections were incubated with appropriate Biotinylated secondary antibodies (1:200) and reacted using the avidin-biotin complex elite kit (Vector Laboratories, Burlingame, CA). The colorimetric TUNEL system (Promega, Madison, WI) was also used to identify apoptotic cell death (24).

### Imaging and tissue analysis

All brain sections were imaged using a slide scanner (Leica Brightfield Aperio ScanScope AT Turbo). Analyses were performed on coded slides (observer blinded to treatment) using either ImageScope (Aperio Technologies, California, USA; NeuN, sheep serum, TUNEL analyses) or ImageJ (NIH image, Bethasda, Maryland, USA; Iba-1 and GFAP analyses). Immunohistochemical analyses were performed on one section from each of the frontal, parietal, temporal and occipital lobes from each lamb; the same regions of gray matter were assessed in each animal. NeuN^+^ neurons were counted in one field of view (FOV; 0.56 mm^2^) in three gyri as previously described (25). Each field was further divided into four bins for the analysis of the different cortical layers (bin 1: cortical layer 1; bin 2: layers 2 + 3; bin 3: layer 4; bin 4: layers 5 + 6). Gray matter area was measured in NeuN labeled sections using Aperio ImageScope software. Gray matter area was determined by measuring the total combined area (mm^2^) from the four bins used to count NeuN^+^ neurons within each cortical layer. Caspase 8^+^ and TUNEL^+^ cells were counted throughout the cortical gray matter. Iba-1^+^ resting (ramified) and activated (amoeboid) microglia (distinguished by morphology) and GFAP-immunoreactive astrocytes were counted in random fields of view throughout the cortical gray matter (12 fields/section/lamb; FOV: 0.140 mm^2^). The total number of vessel profiles with serum albumin extravasation within the cortical gray matter were counted, and expressed as the total number of leaky vessels per entire cortical gray matter area.

### Statistical analysis

Physiological data was analyzed using a 2-way repeated measures ANOVA using treatment (LPS vs. SAL) and ventilation (INJ vs. PROT ventilation) as the variables. The histological data was analyzed using a 2-way ANOVA. *Post-hoc* analysis was conducted using the Holm-Sidak test. All data are presented as mean ± SEM, values of *p* < 0.05 were considered statistically significant.

## Results

### Ventilation and oxygenation

**Figure 1:**
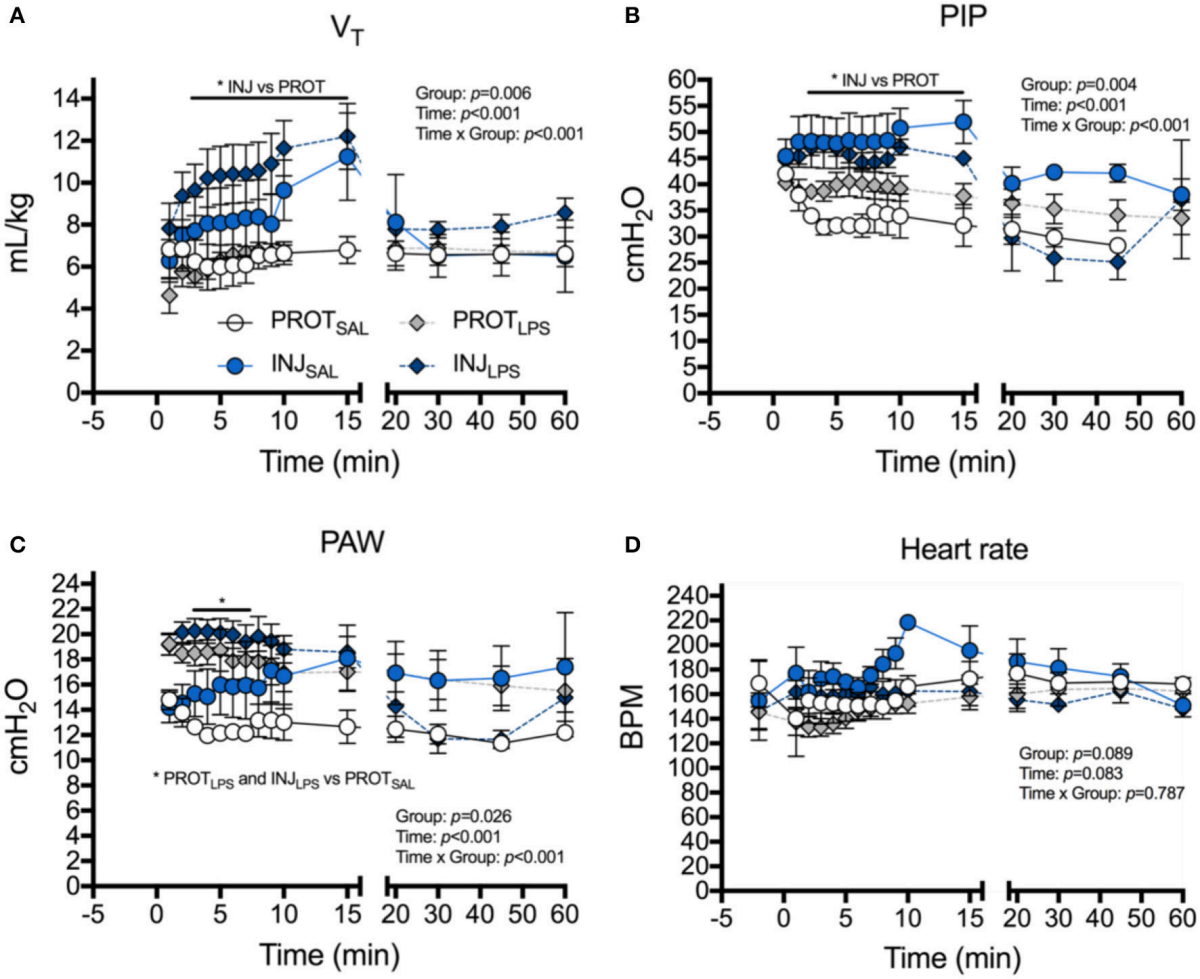
Effects of ventilation strategy and LPS exposure on ventilator requirements and heart rate. Changes in tidal volume (V_T_), **(A)**, peak inspiratory pressure (PIP) **(B)**, mean airway pressure (PAW) **(C)** and heart rate **(D)**, in the PROT_SAL_ (white circles), INJ_SAL_ (blue circles), PROT_LPS_ (gray diamonds) and INJ_LPS_ (dark blue diamonds) lambs. PROT_SAL_, *N* = 5; PROT_LPS_, *N* = 6; INJ_SAL_, *N* = 6; and INJ_LPS_, *N* = 5. ^*^p < 0.05.

Tidal volume was significantly higher from 4 to 15 min in INJ_SAL_ and INJ_LPS_ compared to PROT_SAL_ and PROT_LPS_ (^*^p < 0.05; Figure 1A). There was no difference from 15 min when INJ lambs were placed on the PROT ventilation strategy. Similarly, PIP was significantly higher in the INJ ventilated lambs compared to those receiving the PROT strategy from 3 to 15 min (^*^p < 0.05; Figure 1B).

Overall, group mean PAW tended to be higher in INJ_LPS_ (^*^p < 0.05) and PROT_LPS_ (^*^p < 0.05) lambs compared to PROT_SAL_ lambs. PAW was significantly higher in INJ_LPS_ and PROT_LPS_ lambs at 3–7 min compared to PROT_SAL_ lambs (Figure 1C). Heart rate transiently increased in INJ_SAL_ lambs at 10 min compared to the other groups (Figure 1D) but no other differences in heart rate were observed.

**Figure 2:**
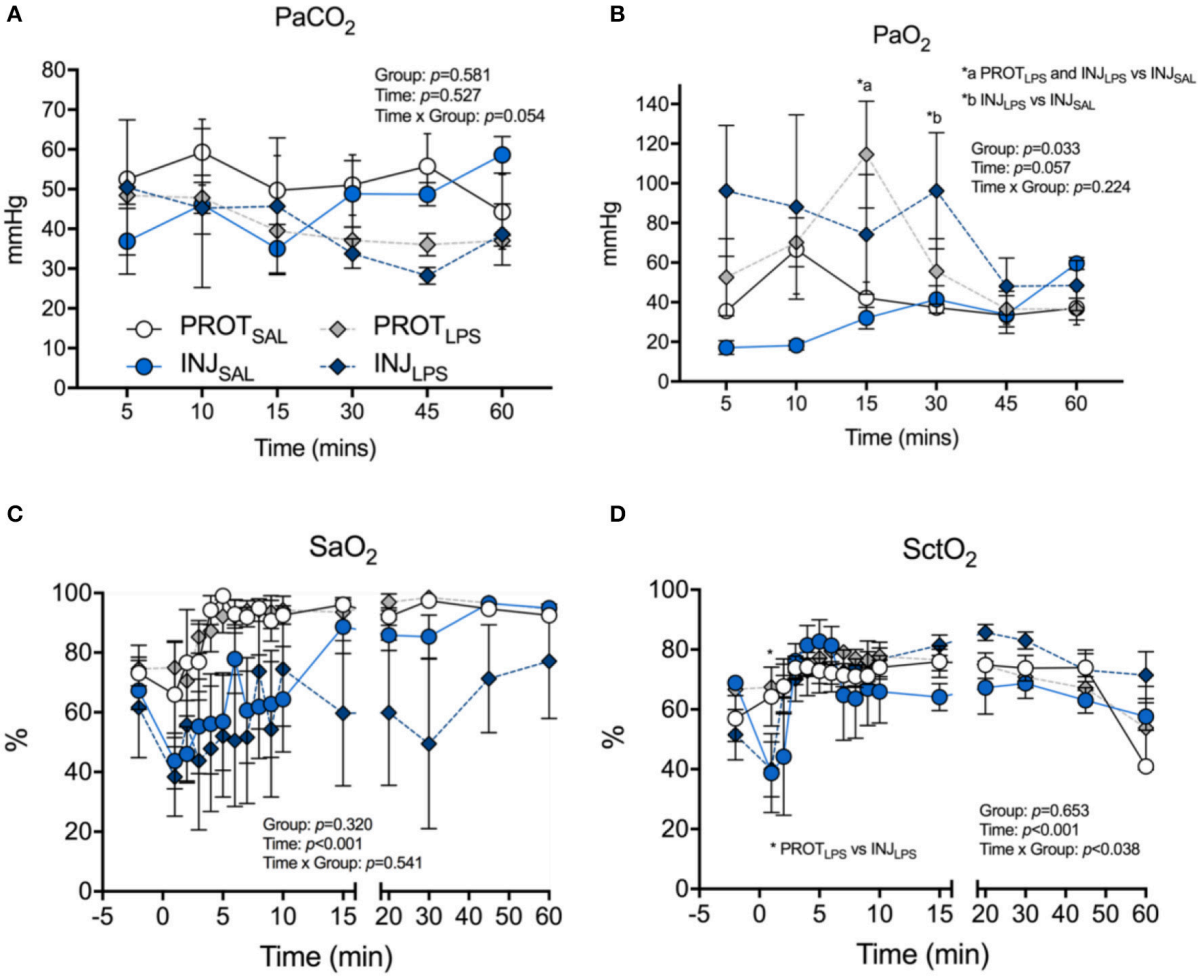
Effects of ventilation strategy and LPS exposure on blood gases and oxygen saturation. Changes in partial pressure of CO_2_ (PaCO_2_) **(A)**, and partial pressure of O_2_ (PaO_2_) **(B)** in arterial blood, arterial oxygen saturation (SaO_2_) **(C)**, and cerebral tissue O_2_ saturation (SctO2) **(D)** in the PROT_SAL_, INJ_SAL_, PROT_LPS_ and INJ_LPS_ lambs. PROT_SAL_, *N* = 5; PROT_LPS_, *N* = 6; INJ_SAL_, *N* = 6; and INJ_LPS_, *N* = 5. *p < 0.05.

PaCO_2_ was not different between groups throughout the ventilation procedure (Figure 2A). PaO_2_ in the INJ_LPS_ group was significantly higher at 15 and 30 min compared to the INJ_SAL_ ventilation cohort (^*^p < 0.05; Figure 2B). Similarly, PaO_2_ was significantly higher in the PROT_LPS_ group at 15 compared to the INJ_SAL_ group at 30 min (^*^p < 0.05; Figure 2B). No other differences were observed. SaO_2_ was not different between the groups throughout the ventilation procedure (Figure 2C). SctO_2_ was lower in INJ_LPS_ lambs compared to the PROT_LPS_ group at 1 min (^*^p < 0.05; Figure 2D) but no other differences were observed.

### Neuronal density

**Figure 3:**
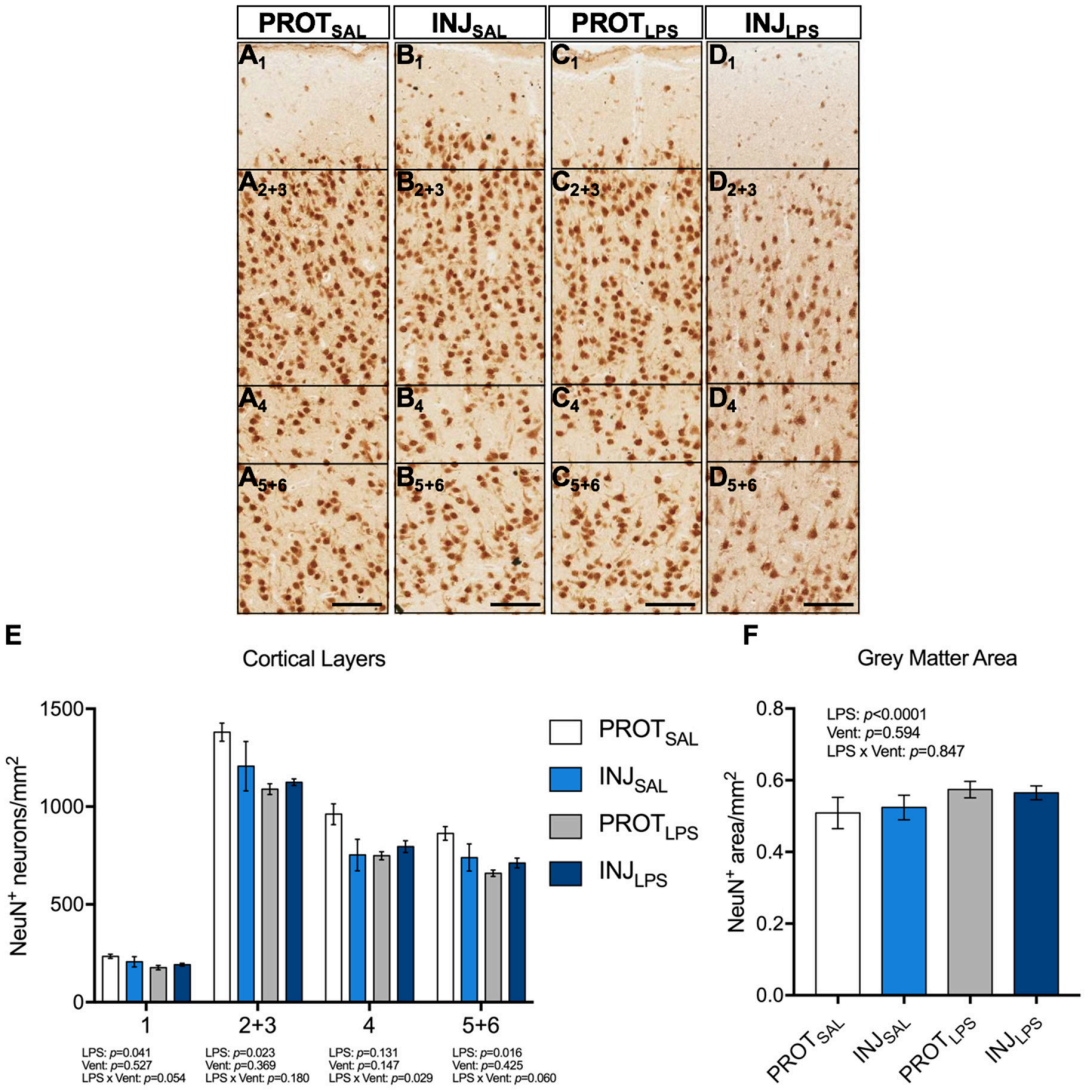
LPS exposure, irrespective of ventilation strategy leads to neuronal loss in the cortical layers and is associated with changes in gray matter area. Representative images of NeuN^+^ neurons in the cortical layers of the gray matter **(A**_1_**-D**_5+6_**)**. LPS exposure induced significant neuronal loss throughout all cortical layers (except 4) **(E)**. Gray matter area was significantly higher in the LPS-treated groups (*p* < 0.0001) when compared to the saline-treated lambs, whilst ventilation had no effect **(F)**.Magnification = 2x, scale bar = 20 μm. PROT_SAL_, *N* = 5; PROT_LPS_, *N* = 6; INJ_SAL_, *N* = 6; and INJ_LPS_, *N* = 5.

The number of NeuN^+^ neurons from all 6 cortical layers were counted (total 1 mm^2^ area) (Figures 3A–D). A significant reduction in the number of neurons in all cortical layers except 4 was observed in LPS lambs compared to controls (layer #1: *p* = 0.041; layers #2 + 3: *p* = 0.023; layers #5 + 6: *p* = 0.016) (Figure 3E). No significant differences in the number of NeuN^+^ neurons in cortical layer #4 were observed (Figure 3E). Furthermore, gray matter area was significantly higher in the LPS-treated groups (*p* < 0.0001) when compared to the saline-treated lambs, whilst ventilation had no effect (Figure 3F).

### Apoptotic cell death

**Figure 4:**
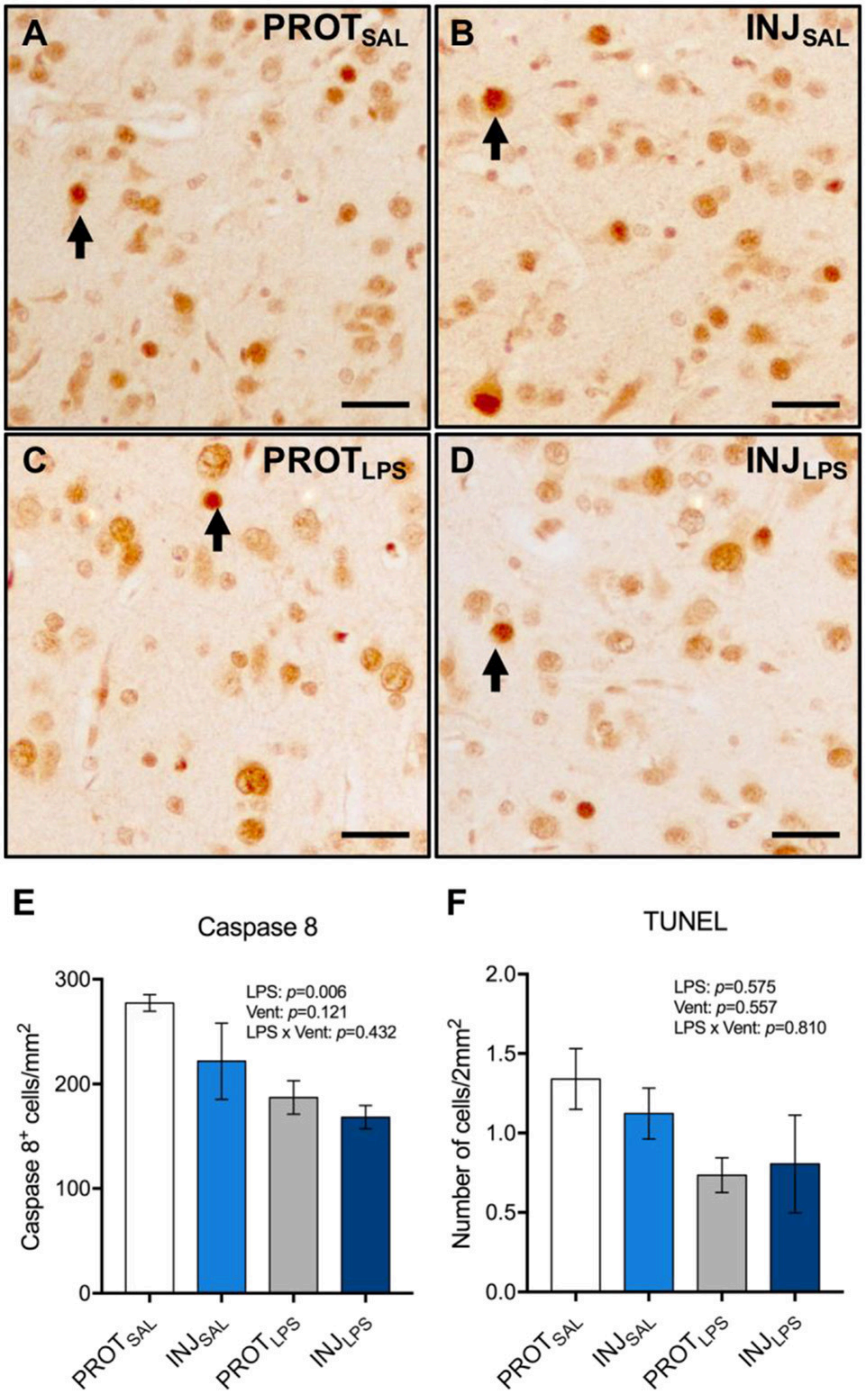
Ventilation strategy and LPS exposure did not induce apoptosis in cortical gray matter. Representative images of Caspase 8^+^ cells in the gray matter **(A–D)**. A significant reduction in the number of Caspase 8^+^ cells was observed in the LPS-treated groups compared to the respective saline controls **(E)**. No significant differences in the number of TUNEL^+^ cells were observed between the groups **(F)**. Magnification = 8x, scale bar = 20 μm. PROT_SAL_, *N* = 5; PROT_LPS_, *N* = 6; INJ_SAL_, *N* = 6; and INJ_LPS_, *N* = 5.

The number of Caspase 8^+^ cells in the cortical gray matter was significantly reduced in the PROT_LPS_ and INJ_LPS_ cohorts (*p* = 0.006; *N* = 5/6) (Figures 4A–E). No significant differences in the number of TUNEL^+^ cells were observed between the groups (Figure 4F).

### Microglia

**Figure 5:**
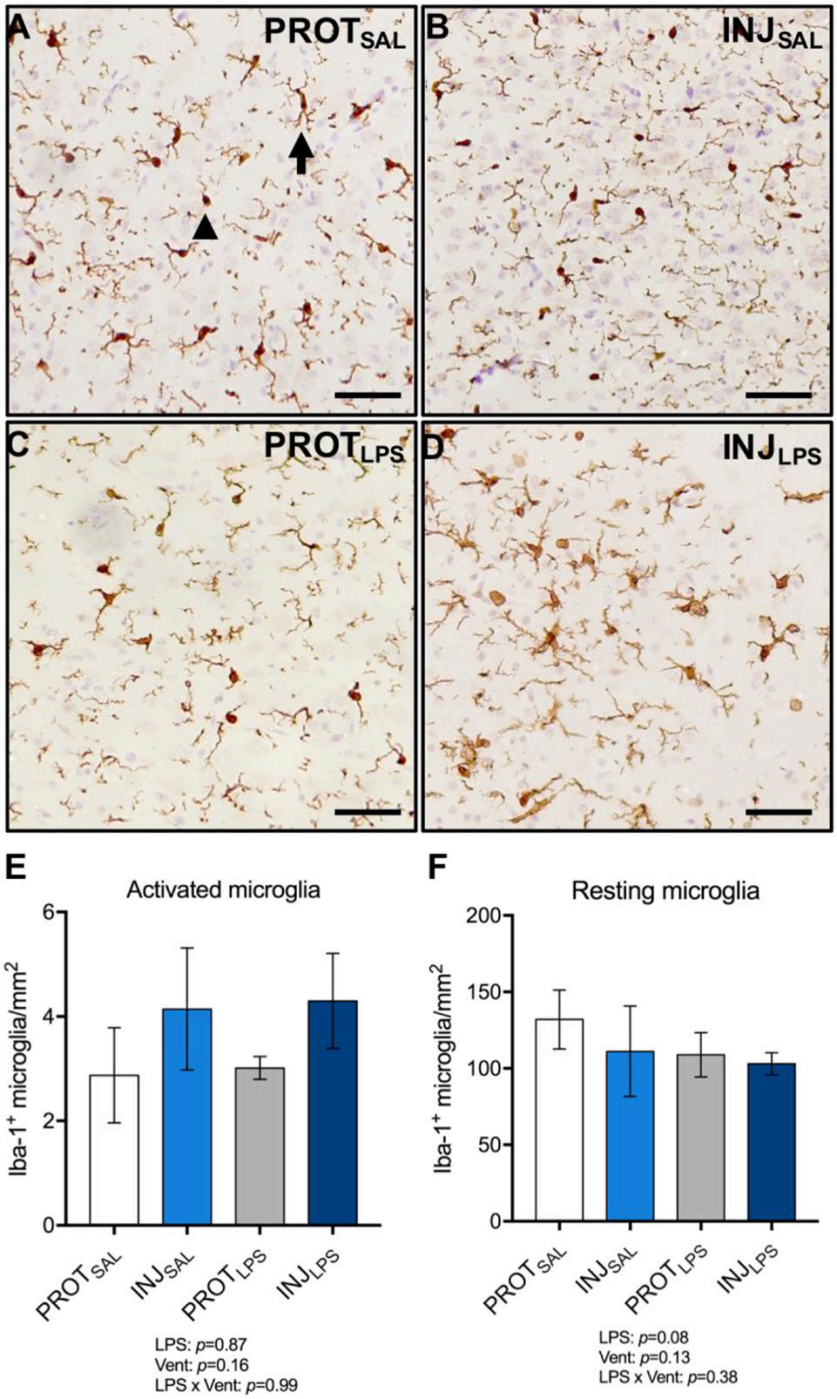
LPS exposure, irrespective of ventilation strategy, does not change the number of activated or resting microglia in the cortical gray matter. Representative images of Iba-1^+^ microglia in the gray matter **(A–D)**. LPS exposure did not significantly change the number of activated (amoeboid; arrowhead) microglia **(E)**, or resting (ramified; arrow) microglia **(F)**. Magnification = 2x, scale bar = 20 μm. PROT_SAL_, *N* = 5; PROT_LPS_, *N* = 6; INJ_SAL_, *N* = 6; and INJ_LPS_, *N* = 5.

To determine the effect of LPS and ventilation strategy on microglial activation, Iba-1^+^ cells from the cortical gray matter were counted (total 1 mm^2^ area) (Figures 5A–D). Microglia showing ramified dendritic processes were considered to be in a resting/quiescent state, and those displaying retractive and thickened processes and/or are amoeboid in shape are considered to be activated. No significant differences in the number of activated microglia were observed between groups (LPS: *p* = 0.87; vent: *p* = 0.16; LPS × vent: *p* = 0.99) (Figure 5E). No significant differences in the number of resting microglia were observed between the treatment groups (LPS: *p* = 0.08; vent: *p* = 0.13; LPS × vent: *p* = 0.38) (Figure 5F).

### Astrocyte number

**Figure 6:**
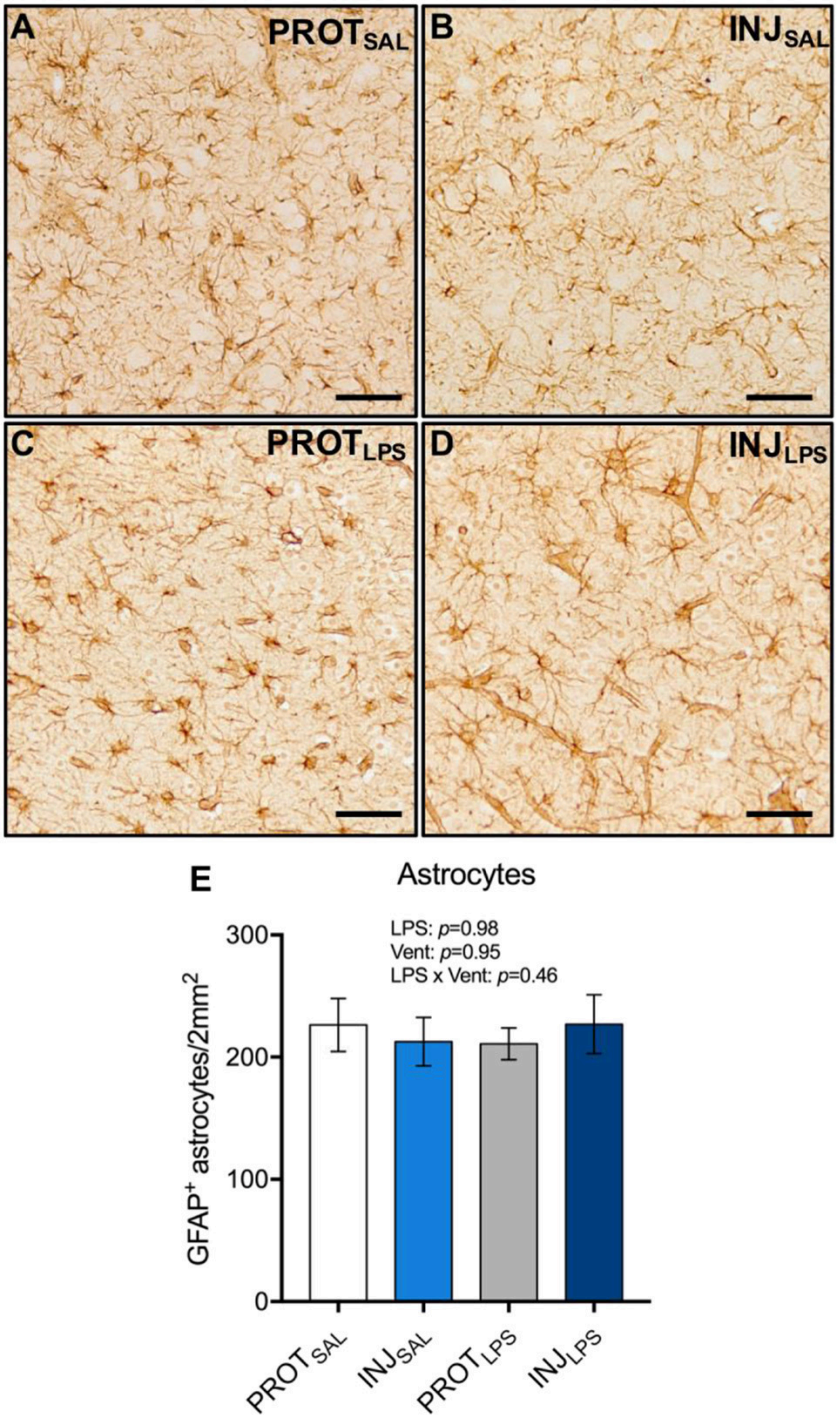
Ventilation strategy and LPS exposure does not alter astrocyte numbers in the cortical gray matter. Representative images of GFAP^+^ astrocytes in the gray matter **(A–D)**. Neither ventilation strategy, or LPS exposure caused changes in the number of astrocytes **(E)**. Magnification = 2x, scale bar = 20 μm. PROT_SAL_, *N* = 5; PROT_LPS_, *N* = 6; INJ_SAL_, *N* = 6; and INJ_LPS_, *N* = 5.

To determine whether PROT or INJ ventilation, or LPS exposure induces astrogliosis, GFAP^+^ astrocytes from the cortical gray matter were counted (total 1 mm^2^ area) (Figures 6A–D). No significant differences in astrocyte numbers were observed between all groups (LPS: *p* = 0.98; vent: *p* = 0.95; LPS × vent: *p* = 0.46) (Figure 6E).

### Blood brain barrier permeability

**Figure 7:**
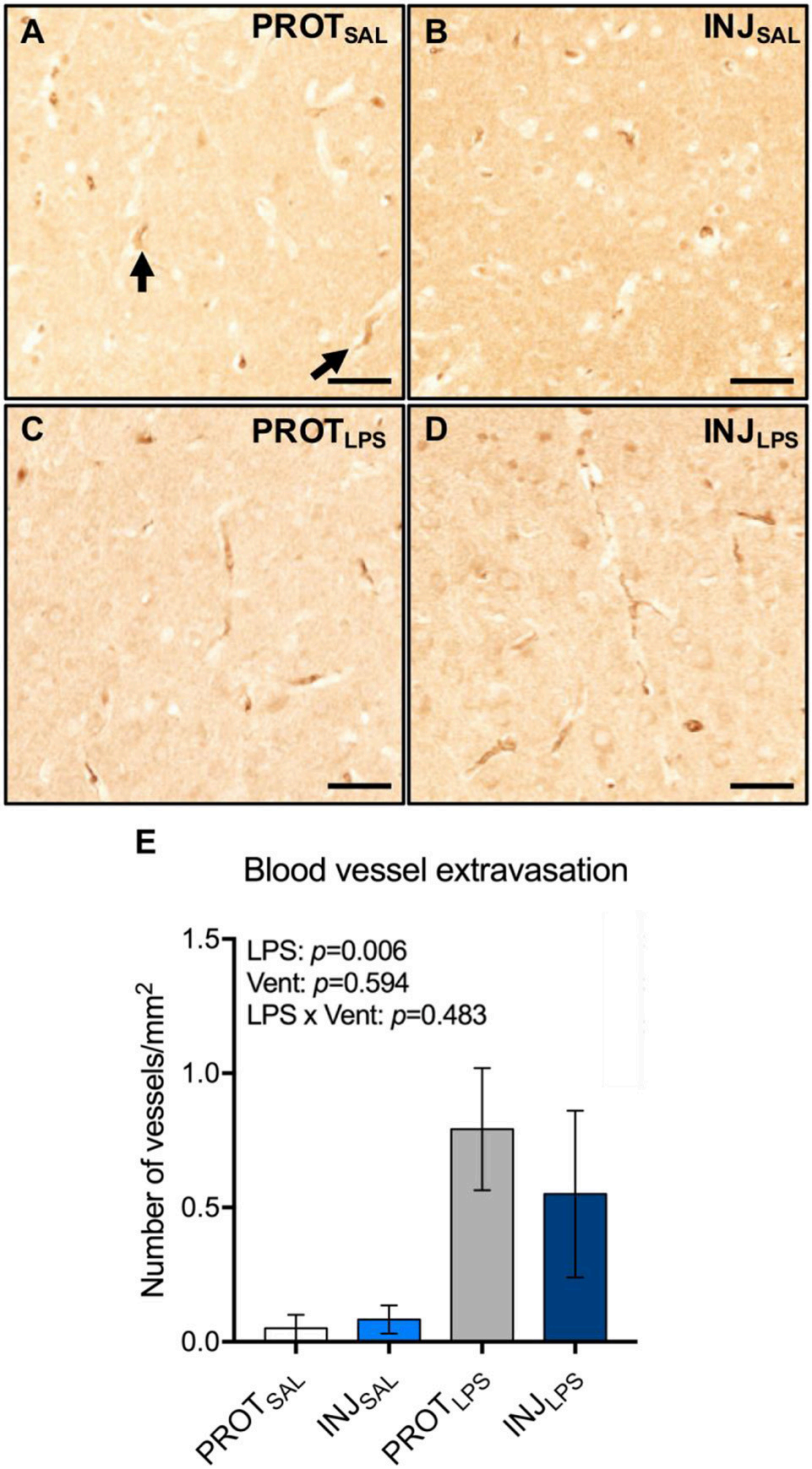
LPS exposure is associated with vessel protein extravasation in the cortical gray matter. Representative images of blood vessels labeled for sheep serum in the gray matter **(A–D)**. LPS exposure leads to an increase in the number of blood vessels with protein extravasation **(E)**. Magnification = 4x, scale bar = 20 μm. PROT_SAL_, *N* = 5; PROT_LPS_, *N* = 6; INJ_SAL_, *N* = 6; and INJ_LPS_, *N* = 5.

The total number of blood vessel profiles with protein extravasation in the cortical gray matter were counted (Figures 7A–D). LPS treatment was associated with blood vessel protein extravasation (LPS: *p* = 0.006), whereas ventilation alone or LPS combined with ventilation were not associated with a significant increase in extravasation (vent: *p* = 0.594; LPS × vent: *p* = 0.483) (Figure 7E).

## Discussion

Respiratory support is a life-sustaining intervention for preterm neonates that cannot breathe sufficiently or autonomously at birth, however, if delivered inappropriately, can cause preterm white matter injury (3, 5, 6, 9). In addition, ventilation after chorioamnionitis exacerbates white matter inflammation and injury (7, 9). However, the effect on the gray matter is not known. In this study, we found that LPS exposure, irrespective of ventilation strategy, caused gray matter edema, reduced neuronal cell density throughout the cortical layers and increased vascular protein extravasation. While injurious ventilation tended to increase activated microglial number, no discernible effect of ventilation strategy was observed. These findings suggest that improving ventilation of preterm infants exposed to chorioamnionitis may not reduce gray matter inflammation and injury.

We have previously demonstrated that injuriously high tidal volumes initiate white matter inflammation and injury via two mechanisms; systemic inflammation and haemodynamic instability (7, 9). In this study, V_T_ was significantly higher in injuriously ventilated groups compared to gentle ventilation within the first 15 min. LPS-exposed lambs had a higher PAW compared to the PROT_SAL_ cohort. This indicated better compliance initially, but this effect was limited to 4 min within the initial 15 min of ventilation. Furthermore, it should be noted that PaO_2_ was significantly higher in the LPS-treated groups compared to lambs that received saline. Exposure to LPS/intrauterine inflammation causes thinning of the tissue-airspace barrier resulting in improved gas exchange initially after birth (26, 27). This results in improved PaO_2_ at the same inspired oxygen.

White matter brain injury has been reported in preterm infants exposed to intrauterine infection and inflammation (7, 14, 28–30). Injurious high V_T_ ventilation increases neuroinflammation, protein extravasation, and lipid peroxidation in the white matter of preterm lambs. Further, LPS exposure exacerbates periventricular white matter injury, irrespective of the ventilation strategy used (PROT or INJ). Indeed, LPS exposure upregulates IL-6 and IL-8 cytokine expression within the white matter, and is associated with increased microglial activation, astrogliosis, apoptosis, and protein extravasation (7). Whilst no significant differences in the number of activated microglia were observed, the immunohistochemical labeling shows clear structural remodeling of microglia (amoeboid morphology) adjacent to those with a resting, ramified phenotype in the gray matter, which would account for the high variability in the counts. Our results are in contrast to ventilation and LPS-induced white matter injury, in which the underlying mechanism of damage is thought to be severe microglial-induced inflammation. Nevertheless, our data suggest that injurious ventilation had little effect on acute cortical gray matter inflammation and injury in the late preterm brain. A recent study in preterm infants has shown cortical gray matter injury using MRI, and subsequent lower cognitive scores (31). Prolonged mechanical ventilation was associated with a higher rate of brain abnormalities on term equivalent age MRI. Thus, while the initial resuscitation strategy may not produce immediate effects within the cortical gray matter, prolonged ventilation appears to have adverse consequences for the preterm brain, which include but are not limited to cortical gray matter.

It is possible that VIBI within the cortical gray matter takes longer than 2 h to manifest. By contrast we have previously demonstrated that this time period is sufficient for the development of inflammation and injury within the white matter (7, 9). Further studies are required to determine whether gray matter inflammation and injury is present days after the initial resuscitation insult. Furthermore, it should be noted that infants that receive prolonged ventilatory support would have many confounding factors that may contribute to brain injury.

In this study, LPS treatment caused a significant reduction in the number of NeuN^+^ neurons in all cortical layers, except layer 4. Neuronal loss and reduced gray matter volumes have been observed in preterm infants that received prolonged respiratory support and/or were exposed to intrauterine inflammation (32–39). Whilst the mechanisms of neuronal death and changes in gray matter volumes have not been elucidated, it is thought that they may have some overlap with white matter injury. Furthermore, we observed reduced apoptosis in the LPS-exposed cohorts. However, the decrease in apoptosis simply reflects the increased cortical gray matter area in the LPS-treated groups, indicative of edema. The larger area would reduce cell density if the same number of cells were present.

Neither ventilation strategy or LPS exposure led to significant changes in the number of astrocytes in the cortical gray matter. There are limited data regarding astrogliosis within the cortical gray matter following chorioamnionitis. However, similar to microglia, astrocytes can modulate inflammatory responses within the brain, particularly through their ability to participate in pro-inflammatory cytokine production (40). The lack of astrocytosis following ventilation or LPS exposure suggests other mechanisms mediate cortical neuronal injury. Moreover, LPS-exposure was associated with an increase in blood vessel protein extravasation, irrespective of the ventilation strategy. Albumin is the most abundant protein found in blood serum and is not found outside of cerebral blood vessels. However, cerebral blood vessels may become leaky resulting in serum/protein extravasation. This is a known marker of compromised blood brain barrier integrity (7, 9).

This study has some limitations in translation, particularly due to the model being used. While preterm lambs at 126 days gestation have relatively immature lungs and require respiratory support in order to survive, their brains are relatively mature, and more accurately reflect a near-term infant (41). Indeed, gray matter injury, as detected by MRI in term infants, is likely to be more prominent after severe insults, such as moderate-to-severe asphyxia/hypoxic injury (42). Thus, it may be that the insult from ventilation does not cause overt cortical injury in the late-preterm brain acutely after birth. We did not assess maturation of the neurons in this study. Previous studies in humans and sheep have shown maturational arrest of oligodendrocytes and neurons is a hallmark of preterm brain injury (43). However, it is unlikely that a brief ventilatory period would alter neural maturation. Finally, the ventilator insult, although pronounced, was only brief (15 min). Prolonged respiratory support has been associated with white and gray matter preterm brain injury (31). Thus, it is possible that longer ventilation would be associated with greater cortical injury.

In summary, injurious ventilation had little effect on gray matter inflammation or injury. LPS-exposure increased neuronal loss, edema and protein extravasation within the cortical gray matter. Neuronal loss was not accompanied by increased gliosis, which is typically observed in white matter brain injury following mechanical ventilation or chorioamnionitis. These data suggest that the underlying mechanisms and/or timing of cortical gray matter injury following intrauterine inflammation and ventilation may differ from white matter injury.

## Author contributions

VS wrote manuscript, analyzed data; AA assisted with data collection analysis and contributed to manuscript writing; IN, BS, SKB, VZ, and KR assisted with animal work; SKB, VZ, and SBH revised the manuscript and contributed to data interpretation. GRP and RG were responsible for study conception, design, manuscript revision and oversight of the research.

### Conflict of interest statement

The authors declare that the research was conducted in the absence of any commercial or financial relationships that could be construed as a potential conflict of interest.
